# List of Officers and Committees for 1866

**Published:** 1865-07

**Authors:** 


The Committee on Nomination reported that the time of the next meeting shall be in June, and submitted the following list of officers:—

*President*, Dr. D. H. Storer, of Massachusetts, *Vice-Presidents*, Drs. James F. Hibberd, of Indiana; S. O. Almy, of Ohio; T. C. Dunn, of Rhode Island; W. P. Johnson, of the District of Columbia; *Assistant-Secretary*, Dr. J. E. Morgan, of Maryland.

*Committee on Arrangements*, Drs. C. C. Cox, W. C. Van Bibler, Franklin Donelson, L. H. Steiner, George G. Miltenberger, William Whitridge, J. E. Morgan, all of Baltimore.

*Committee on Publication*, Drs. F. G. Smith, of Pennsylvania; H. F. Askew, of Delaware; William Mayburry, of Pennsylvania; W. B. Atkinson, of Pennsylvania; H. R. Storer, of Massachusetts; Caspar Wister, of Pennsylvania.

*Committee on Prize Essays*, Drs. Austin Flint, senior, J. R. Wood, Ellsworth Elliot, E. Krackowitzer, D. C. Enos, all of New York.

*Committee on Medical Education*, Drs. S. D. Gross, of Pennsylvania; G. P. Twitchell, of New Hampshire; C. A. Pope, of Missouri; O. W. Holmes, of Massachusetts; Grafton Tyler, of District Columbia.

Medical Literature (present committee continued over), Dr. Charles A. Lee, of New York; T. F. Rochester, of New York; C. C. Cox, of Maryland; Albert Smith, of New Hampshire; A. Nebinger, of Pennsylvania.

*Committee on American Necrology*, Drs. C. C. Cox, of Maryland; E. B. Stevens, of Ohio: W. F. Peck, of Iowa; H. Van Dusen, of Wisconsin; Noble Young, of the District of Columbia; Josiah Simpson, of the United States Army; J. C. Weston, of Maine; Henry Bronson, of Connecticut; Henry Noble, of Illinois; Charles Eversfield, of the United States Navy; William B. Fletcher, of Indiana; I. C. Hupp, of Western Virginia; J. Mauran, of Rhode Island; William K. Bowling, of Tennessee; J. P. Finch, of New Hampshire; James Couper, of Delaware; W. L. Linton, of Missouri; Charles L. Allen, of Vermont; H. G. Clark, of Massachusetts; J. H. Griscom, of New York; E. M. Moore, of New York; Charles A. Logan, of Kansas; William B. Little, of California; Stuart, of Minnesota; Henry Miller, of Kentucky; S. G. Armor, of Michigan; William Pierson, of New York; Fleming, of Pennsylvania; E. Wallace, of Penn.

The report was adopted as a whole, with the exception of the time of holding the next annual meeting. It was voted to meet on the first Tuesday in May, 1866, instead of June.

Dr. Allen, of New York, with a few explanatory remarks, moved that the vote adopting the resolution expelling Dr. Pallen be reconsidered. A sharp discussion ensued, and the vote being taken, the result was doubted. The yeas and nays were then called for by Dr. Sayre, of New York, so that the names of the gentlemen might appear on the record. The question being put, "Shall the yeas and nays be taken," was lost. A standing vote was taken, and the motion to reconsider was also lost.

